# Searching for separation between frequency-modulated calls of blue and fin whales

**DOI:** 10.1038/s41598-026-71077-1

**Published:** 2026-09-15

**Authors:** Svenja Wöhle, Clea Parcerisas, Susannah Buchan, John Calambokidis, Kathleen M. Stafford, Elena Schall

**Affiliations:** 1 Alfred-Wegener-Institute Helmholtz Center for Polar and Marine Research, 27570 Bremerhaven, Germany; 2 Flanders Marine Institute (VLIZ), Oostende, 8400 Belgium; 3 IBME, Ghent University, Ghent, 9052 Belgium; 4 Center for Oceanographic Research COPAS COASTAL, Universidad de Concepción, Casilla, Concepción, Región del Biobío Chile; 5 Facultad de Ciencias Naturales y Oceanográficas, Departamento de Oceanografía, Universidad de Concepción, Casilla, Concepción, Región del Biobío Chile; 6 Centro de Estudios Avanzados en Zonas Áridas (CEAZA), Raúl Bitrán, La Serena, Chile; 7 Cascadia Research Collective, Olympia, WA USA; 8 Marine Mammal Institute, Oregon State University, Newport, OR USA

**Keywords:** Ecology, Ecology, Ocean sciences, Zoology

## Abstract

Reliable differentiation of similar acoustic signals remains a major challenge for passive acoustic monitoring (PAM) of sympatric marine mammal species. Blue (*Balaenoptera musculus*) and fin whales (*Balaenoptera physalus*) produce frequency-modulated (FM) downsweeping calls with overlapping frequency ranges and durations, making species attribution difficult. This study combines long-term PAM datasets with animal-borne tag recordings to investigate how these FM calls can be reliably distinguished by species. Tag recordings with confirmed species identity showed substantial overlap in most acoustic features, but the parameter Duration 90% offered partial separation: with most fin whale FM calls < 0.6 s, and blue whale FM calls > 0.7 s. Applied to high-quality calls from moored data with signal-to-noise ratios above 12 dB, this approach may provide a conservative, rule-based means of classification. A more robust approach was achieved by combining deep learning feature extraction with non-linear dimensionality reduction. Integrating confirmed ground-truth tag recordings as a reference space enabled previously unseen FM calls from long-term PAM recordings to be attributed by acoustic similarity to known calls while maintaining an explicit measure of uncertainty by leaving acoustically ambiguous calls unassigned. Together, these approaches provide a practical framework for more reliable call attribution, enhancing long-term PAM-based monitoring of blue and fin whales.

**Supplementary Information:**

The online version contains supplementary material available at https://doi.org/10.1038/s41598-026-71077-1.

## Introduction

Acoustic signals produced by marine mammals can contain important information related to species identity, acoustic population structure, individual identity and behavioral context. Variations in signal properties, such as duration, bandwidth, and frequency range, as well as their overall spectro-temporal patterns in spectrograms, often allow insights into the previously mentioned biological aspects^1–5^.

While passive acoustic monitoring (PAM) technologies are continuously improving, allowing data collection over large spatial- and temporal scales^4^, a potential challenge remains - the reliable differentiation of acoustic signals among species. A similarity in signal parameters and visio-acoustic appearance of calls among sympatric species causes difficulties in accurate species identification^6^. For example, a high-intensity vocalization termed “gunshot call” was originally attributed exclusively to right whales (*Eubalaena spp*.)^7^, but has since been proposed to be produced by humpback whales (*Megaptera novaeangliae*) as well^8^. Humpback whales and right whales also produce another similar vocalization type, the upcall, a common contact call in both species, which has likewise been reported as difficult to distinguish with certainty^6^. Although the acoustic features of upcalls show considerable overlap between the two species, recent analyses suggest that they can be reliably distinguished based on specific acoustic metrics^9^. Another example reported as difficult to distinguish in marine passive acoustic recordings involves the frequency-modulated downsweep vocalizations of baleen whales, particularly blue (*Balaenoptera musculus*), fin (*Balaenoptera physalus*), and sei whales (*Balaenoptera borealis*)^10^. All three species produce downsweeps, also referred to as frequency-modulated (FM) calls, which are non-song vocalizations likely serving a communicative function, such as coordinating group foraging^11–14^. FM calls of blue, fin and sei whales exhibit similar frequency ranges (~ 35–150 Hz) and durations (~ 200 ms to 5s)^10,15^, making species attribution particularly challenging^10,16,17^. Among these species, distinguishing blue and fin whale FM calls is especially problematic given the extensive overlapping their distribution^18,19^.

Although fin and blue whale FM calls, also referred to as 40 Hz-calls^12,20^ and D-calls^21^, respectively, can be difficult to distinguish, the ability to reliably differentiate between them could significantly improve information obtained by PAM efforts. Both species were severely depleted by commercial whaling^22^, and their habitat use, population structures and recovery rates remain poorly understood. Thus, combining the detection of FM calls and blue and fin whale song, which both show only partly overlapping seasonality [e.g.^12,16–18^] would provide a more robust understanding of their occurrence.

Several approaches have been proposed to improve discrimination between similar acoustic signals produced by different whale species. In the case of right and humpback whale upcalls a combination of call parameters, such as slope and bandwidth, has been proven effective^9^. When call parameters overlap substantially, contextual data can be taken into account to confirm the species identity, such as the known geographic distribution. However, this is often not feasible, as multiple species may co-occur within the same geographic region. In such cases, additional cues, such as co-occurring well-known species-specific vocalizations or characteristic call patterns (e.g., sei whale FM calls are supposed to occur in pairs or triplets) may support the decision process for species attribution^14^.

**Fig. 1:**
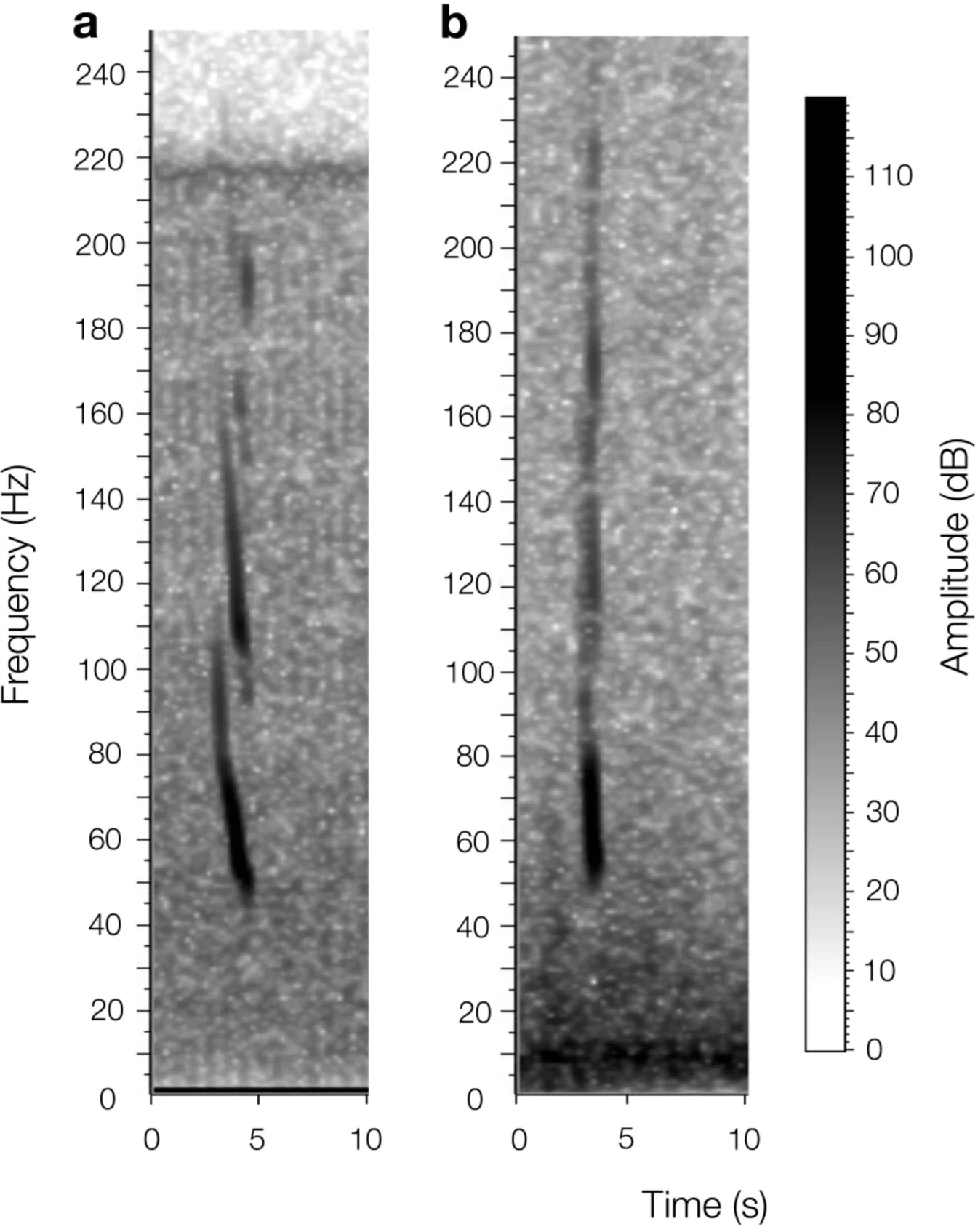
Spectrograms of (**a**) blue and (**b**) fin whale FM calls with multiple harmonics, illustrating their characteristic and highly overlapping frequency contours and ranges. Calls recorded on animal-borne acoustic tags off California in 2012 and Chile in 2024, respectively. Smoothed spectrograms calculated in a Hanning window with FFT/DFT of 512, and 80% overlap.

To date, species differentiation between fin and blue whale FM calls, has primarily relied on differences in call duration^15^, the presence of other species-specific vocalizations co-occurring within a defined time window around the FM call^24^, or automated classification approaches based on spectro-temporal call features^10^. Representative examples of blue and fin whale FM calls are shown in Fig. 1. However, these approaches have never been proven to be reliable, as they have not been validated using species-confirmed ground-truth data and, thus, can limit the possibilities, both spatially and temporally, for attributing calls.

In this study, we aim to explore acoustic differences between blue and fin whale FM calls, with the intention of determining which framework works best for species attribution of FM calls in long-term PAM data. As part of this process, we incorporate species-confirmed ground-truth data from animal-borne tags as a reference to assess whether acoustic parameters, such as call duration, enable reliable differentiation between species.

## Results

### Datasets and labelling

**Fig. 2:**
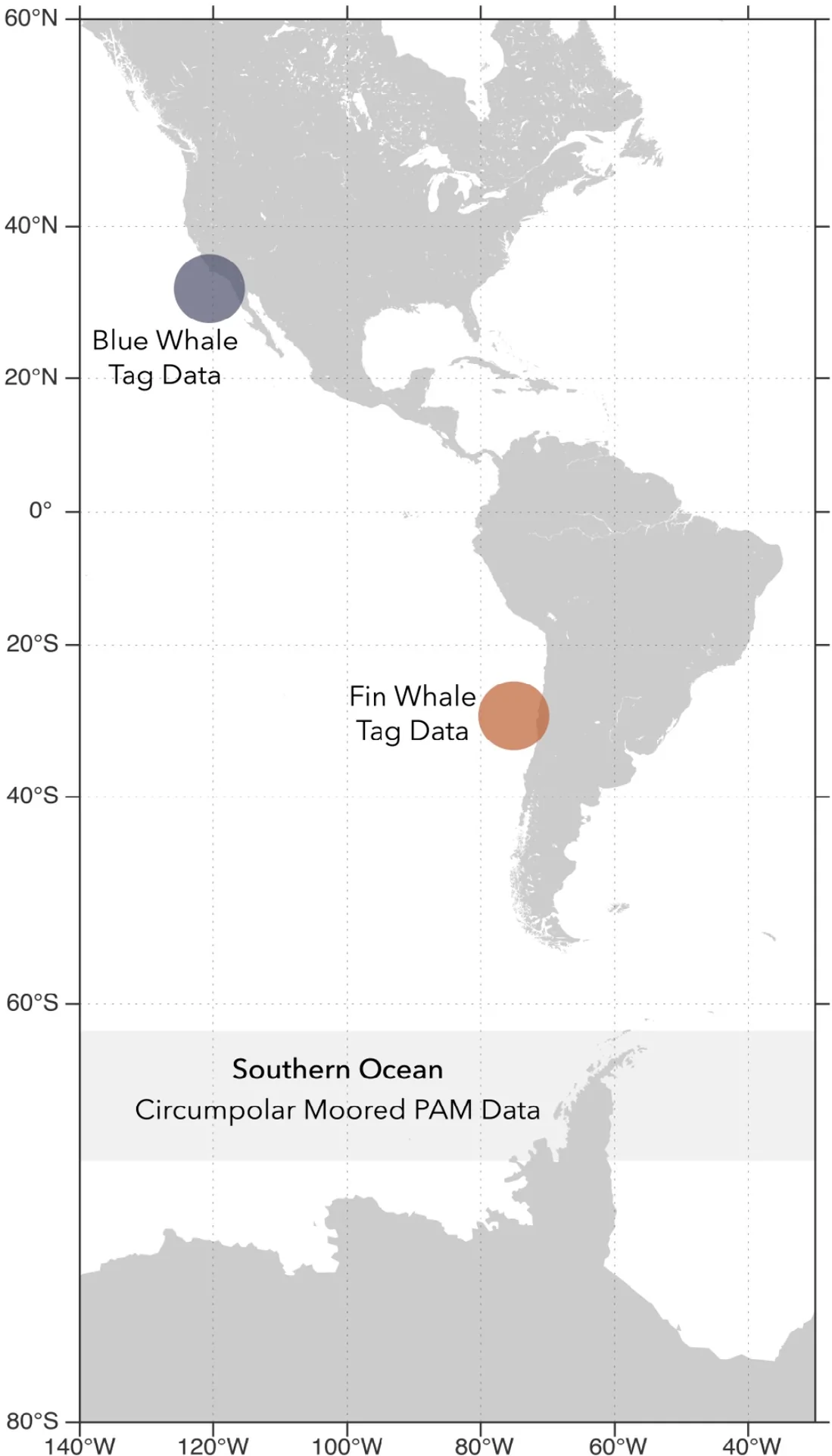
Geographic overview of the datasets used in this study. Blue whale tag recordings were collected off California (blue), and fin whale tag recordings off Chile (orange). The moored PAM dataset was obtained from multiple circumpolar mooring locations in the Southern Ocean (light grey); for detailed mooring positions, see Fig. 10 in the Methods section.

To explore potential differences in call parameters among blue and fin whale FM calls, we analyzed a large passive acoustic dataset from multiple mooring sites across the Southern Ocean as well as a smaller ground-truth dataset from tagging studies off California and Chile (see Fig. 2). The mooring dataset provided broad temporal and spatial coverage but lacked ground-truth information required to reliably assign FM calls to fin or blue whales; therefore, all calls were treated collectively as FM calls. In contrast, the tag dataset contained only a limited number of calls but provided direct confirmation of species, enabling exploration of differences between known blue whale D-calls and fin whale 40 Hz-calls. Together, these complementary datasets enabled assessment of variation in call parameters, evaluation of context-based labelling approaches, and testing of whether tag data can serve as a reference for attributing call types in mooring recordings.

From the mooring recordings, a high signal-to-noise ratio (SNR) Mooring-dataset was curated from the full set of detected FM calls to improve data quality by reducing the influence of low-SNR calls. Excluding all calls with an SNR approximation below 12dB (Raven Pro 1.6 parameter SNR NIST Quick (dB) > 12; The Cornell Lab of Ornithology, Center for Conservation Bioacoustics in Ithaca, NY), this dataset comprised 8,537 out of 18,203 FM calls. The 12dB threshold was chosen operationally as a compromise between data quality and sample size, ensuring reliable measurements while retaining a sufficient number of FM calls, as higher thresholds would have excluded too many calls. Complementarily, the ground-truth dataset from the tagging studies, labeled Tag- dataset contained 66 confirmed 40 Hz fin whale calls and 165 confirmed blue whale D-calls. All tag-derived calls exhibited high signal quality, with a minimum SNR NIST Quick of 14 dB.

### Exploratory data analyses and classification approaches

#### Duration-based classification approach

**Fig. 3:**
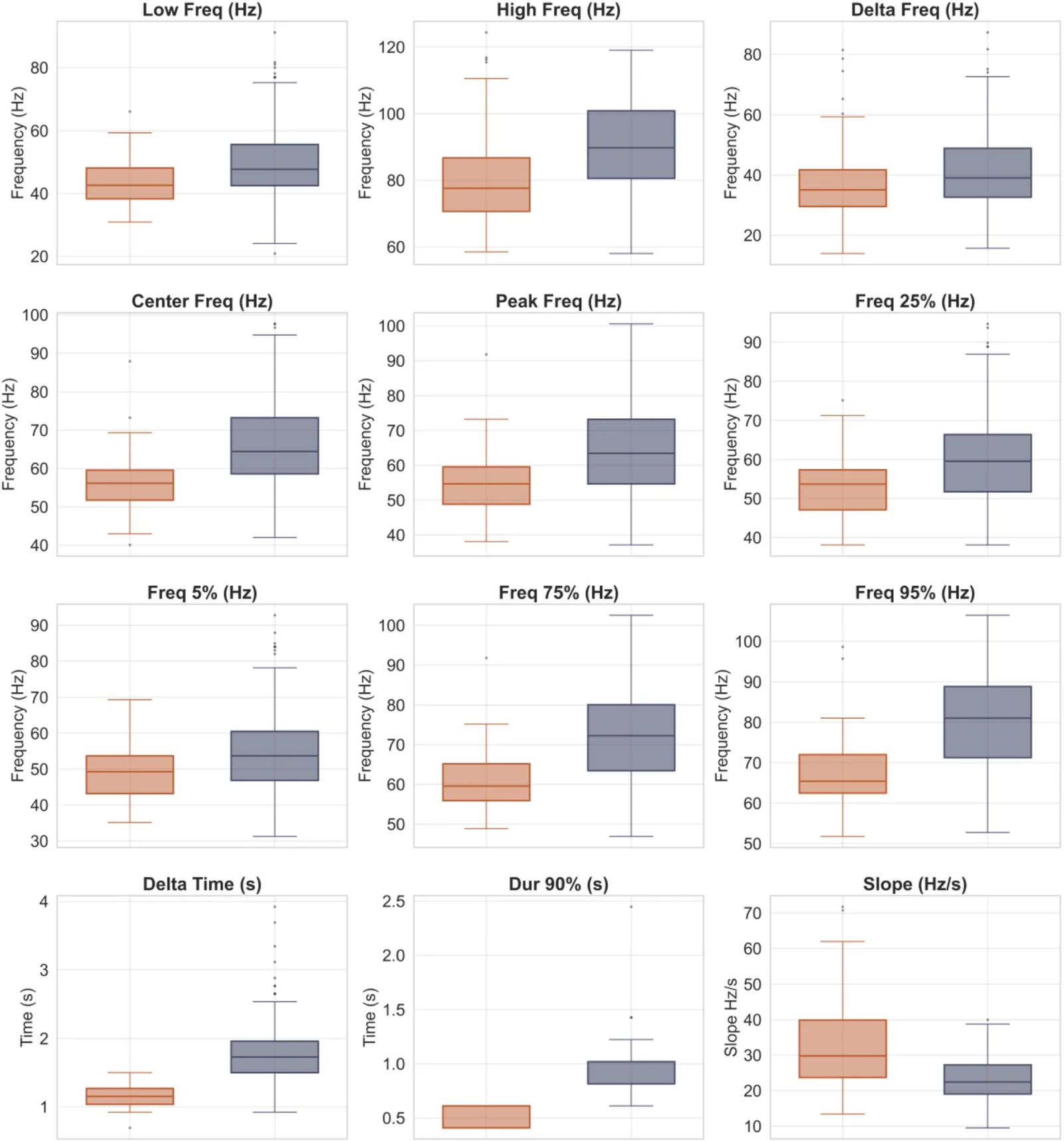
Boxplots representing the 12 quantitative acoustic measurements from the Tag-RavenFeatures dataset for the two confirmed call types: 40 Hz-calls of fin whales (40 - orange), and D-calls of blue whales (*d* - blue).

Previous studies have distinguished blue whale D-calls from fin whale 40 Hz-calls primarily based on call duration, with FM calls longer than approximately 1 - 2 s generally attributed to blue whales and shorter calls to fin whales^15,24^. To evaluate this assumption and assess differences in call characteristics, 12 quantitative acoustic features were extracted from spectrograms of manually annotated calls from the ground-truth tag recordings using RavenPro 1.6 (hereinafter referred to as RavenFeatures). The distributions of these 12 quantitative acoustic features were examined using boxplots for the Tag dataset (Fig. 3), allowing comparison of medians and ranges across call types. In addition, two-sample Kolmogorov–Smirnov tests were used to compare the overall distributions of each acoustic feature between species. Both the boxplots and the Kolmogorov-Smirnov tests revealed largely overlapping distributions of acoustic parameters between D- and 40 Hz-calls, with the strongest distributional separation observed for the duration-based features (Delta Time: KS = 0.845; Duration 90%: KS = 0.842). Call duration (Delta Time), previously proposed as the key parameter for distinguishing blue and fin whale FM calls, nevertheless showed substantial overlap between species in the boxplots. In contrast, Duration 90% is the parameter showing the highest degree of separation, with the exception of a single overlap at 0.612 s, where one call from each species coincides with its 90%-energy call duration. Based on this separation in the ground-truth data, conservative Duration 90% thresholds of < 0.6 s for fin whale 40 Hz calls and > 0.7 s for blue whale D-calls could support differentiation of FM calls. These thresholds were applied to the Mooring-dataset, which could be attributed to 93% of the data. In detail, 3353 calls (39.3%) were attributed to fin whale 40 Hz-calls and 4867 calls (57%) to blue whale D-calls, while 317 calls (3.7%) fall between thresholds and cannot be attributed.

#### Contextual labelling and comparison with call parameters

In addition to call duration, previous studies have also proposed contextual labelling as a means of attributing FM calls to species in long-term moored PAM datasets. Such an approach was applied to the Mooring-dataset to assess its consistency with the Duration 90% classification rule derived from the ground-truth dataset.

The labelling method relied on the presence or absence of well-known species-specific call types within ± 30 min of an FM call. For fin whales, the stereotyped 20 Hz-pulse^25–29^ was used, whereas for Antarctic blue whales the stereotyped Z-call was used^3,29^. To increase the reliability of the labelling, only 20 Hz-pulses and Z-calls with an SNR NIST Quick (dB) > 15 were considered, thereby excluding faint calls likely produced by distant animals. Using this approach, FM calls were attributed as 40 Hz-calls if only fin whale 20 Hz-calls were present in the set time window, as D-calls if only blue whale Z-calls were present, and as frequency-modulated calls (FM; unclassified) if both species or neither species’ calls were present.

**Fig. 4:**
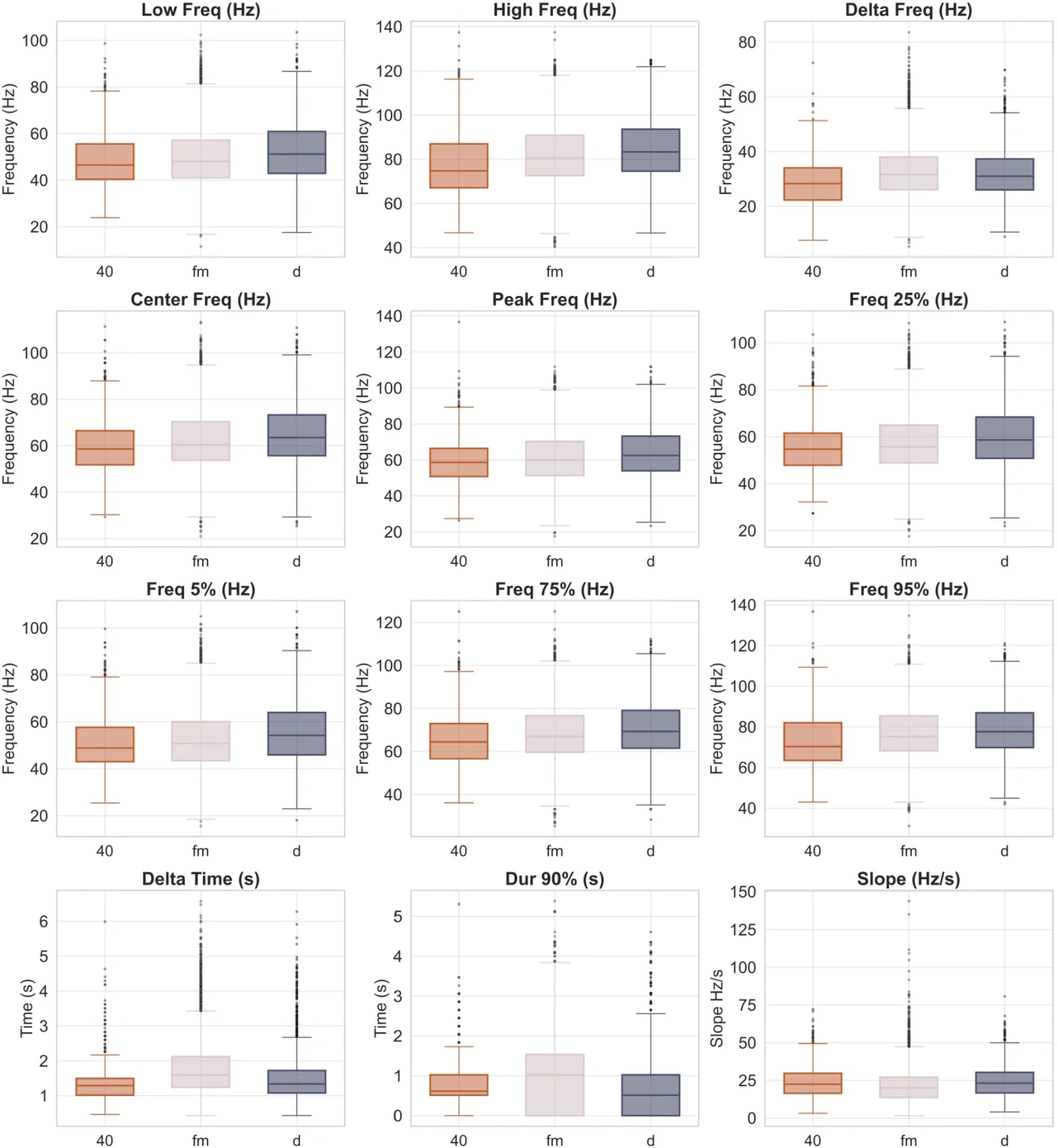
Boxplots representing the 12 quantitative acoustic measurements from the Mooring-dataset for the three groups of contextually labelled FM calls: 40 Hz-calls of fin whales (40 - orange), D-calls of blue whales (*d* - blue), and frequency-modulated (FM) calls of uncertain attribution (fm - light grey).

To evaluate whether contextual labelling is consistent with the Duration 90% rule derived from the ground-truth dataset, both the acoustic feature distributions of contextually attributed call types and the overall agreement between the two classification approaches were examined. Overall, contextual labelling attributed 634 FM calls as fin whale 40 Hz-calls and 1,719 calls as blue whale D-calls, while 6,184 calls remained unclassified. Acoustic differences between contextually attributed call types were further examined using boxplots of Raven features (Fig. 4). Substantial overlap between D- and 40 Hz-calls was observed across most acoustic parameters. Duration 90%, which showed strong separation between species in the ground-truth dataset, also exhibited substantial overlap between contextually attributed D- and 40 Hz-calls. Furthermore, species attributions obtained through both approaches were compared using a confusion-matrix of assigned classes (Table 1). Agreement between the methods was limited. Approximately 45% of contextually attributed fin and blue whale calls received the same classification under the Duration 90% rule. While 6,184 calls remained unclassified under the contextual labelling approach, 6,010 of these could be attributed to either fin whales or blue whales by the Duration 90% rule.

**Table 1:** Confusion-matrix of species attribution from contextual labelling and the Duration 90% rule (ground-truth–based threshold approach). Values show the number of FM calls assigned to each combination of methods. Diagonal elements (bold) indicate matching class assignments between both approaches.

Context label	Duration 90%		
	40 Hz	D-call	FM
40 Hz	**288**	279	67
D-call	861	**782**	76
FM	2204	3806	**174**

### Clustering-based exploration of call structure

As the ground-truth tag dataset comprises a limited number of calls from relatively few deployments, individuals, and acoustic environments, it may not fully capture the variability of FM call structure. Accordingly, it remains uncertain whether the separation observed in the ground-truth data using Duration 90% thresholds is representative of a broader FM call repertoire. To address this, clustering-based analyses were conducted to assess whether call types exhibit separable structure in the overall acoustic feature space using both Raven features and more comprehensive deep learning features (DLFeatures).

Deep learning feature extraction was applied using two pre-trained models, Biolingual^30^, and GoogleWhale^31^. As GoogleWhale performed better overall, only the results obtained with this model are presented in the main text. The corresponding results for Biolingual are provided in the Appendix (Figs. A1–A4 and Tables A1–A4). For both models, audio snippets of the analysed calls were used as input, producing high-dimensional embedding vectors for each call. Each embedding vector represents a learned representation of the spectro-temporal structure of a call and may capture patterns not represented by manually extracted Raven features. Due to some audio snippets being shorter than the required input window for the models, the Mooring-DLFeatures datasets contain 184 fewer data points than the corresponding Mooring-RavenFeatures dataset (8,353 and 8,537 data points, respectively).

#### Clustering Mooring data

**Fig. 5:**
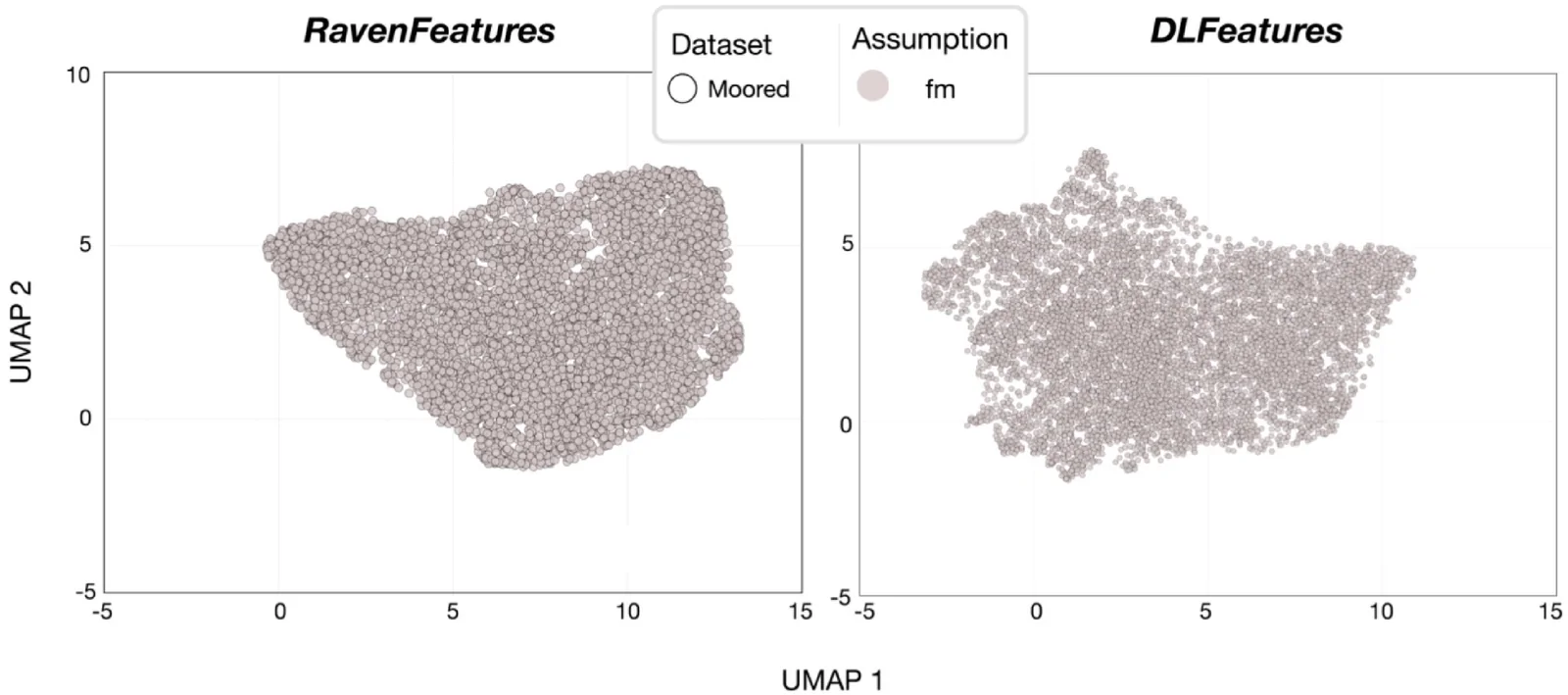
Dimension reduction using an unsupervised UMAP approach on FM calls from the Mooring-RavenFeatures and Mooring-DLFeatures (GoogleWhale).

To explore whether FM calls in the Mooring-dataset exhibit separable structure independent of the Duration 90% and contextual labelling approaches, clustering analyses were first applied to the unlabeled mooring data. A non-linear dimensionality reduction was conducted using a Uniform Manifold Approximation and Projection (UMAP), to explore potential non-linear relationships in the dataset. UMAP was applied to both the RavenFeature vectors and DLFeatures, projecting each call into a two-dimensional space. Under unsupervised conditions, the Mooring-RavenFeature projections form a single broad cluster without distinct separation into discrete groupings (Fig. 5). Across models, UMAP projections of the DLFeatures produce broadly similar patterns. Compared to the RavenFeature projection, the GoogleWhale projection exhibits a more structured and spatially heterogeneous distribution of calls, with several locally denser regions. However, the points remain connected within a continuous manifold rather than forming clearly separated clusters.

To further quantify potential clustering structure, Hierarchical Density-Based Spatial Clustering of Applications with Noise (HDBSCAN), a density-based clustering algorithm, was applied to both the Raven- and the DLFeatures UMAP projection spaces of the Mooring-dataset. Table 2 summarizes the results for the RavenFeatures and the DLFeatures. Overall, the RavenFeatures assigned a larger proportion of calls to clusters, compared to the DLFeatures.

To evaluate cluster quality independently of any external labels, three intrinsic clustering metrics were calculated (Table 2). These metrics assess how well HDBSCAN clusters are separated and how compact they are in the feature space. The identified RavenFeature clusters yield a Silhouette Score of 0.31, a Davies-Bouldin Index of 0.88, and a Calinski-Harabasz Index of 2836.75, indicating moderate clustering structure. In contrast, the DLFeature cluster space achieves the same Silhouette Score (0.31), but a higher Davies-Bouldin Index (1.66), indicating less distinct and less compact clusters compared to the RavenFeature space. Although the Calinski-Harabasz Index is lower for the DLFeatures (702.04), this metric is sensitive to differences in cluster number, size, and distribution and is therefore not directly comparable across clustering solutions with different structures. Overall, the intrinsic metrics suggest weaker clustering structure in the DLFeature space than in the RavenFeature space.

**Table 2:** HDBSCAN clustering results and intrinsic clustering performance metrics for the Mooring-RavenFeature and Mooring-DLFeature-GoogleWhale datasets. The table summarizes the number and proportion of data points assigned to each cluster based on the unknown call types in the Mooring-dataset, together with intrinsic clustering evaluation metrics. Cluster entries show the number of data points, followed by the corresponding percentage (in parentheses) relative to the total number of data points in the respective dataset. Data points classified as noise by HDBSCAN are listed as unassigned. Intrinsic metrics include the Silhouette Score, Davies–Bouldin Index, and Calinski–Harabasz Index, which quantify cluster separation, compactness, and between-cluster dispersion in the feature space, respectively.

Dataset	Cluster	No. total	Silhouette score	Davies-Bouldin index	Calinski-Harabasz index
Mooring-RavenFeatures	Unassigned	776 (9.1%)	0.31	0.88	2826.75
	1	6447 (75.5%)			
	2	1314 (15.4%)			
Mooring-DLFeatures (GoogleWhale)	Unassigned	6073 (72.7%)	0.31	1.66	702.04
	1	67 (0.8%)			
	2	538 (6.4%)			
	3	1675 (20.1%)			

#### Clustering Ground-truth data

**Fig. 6:**
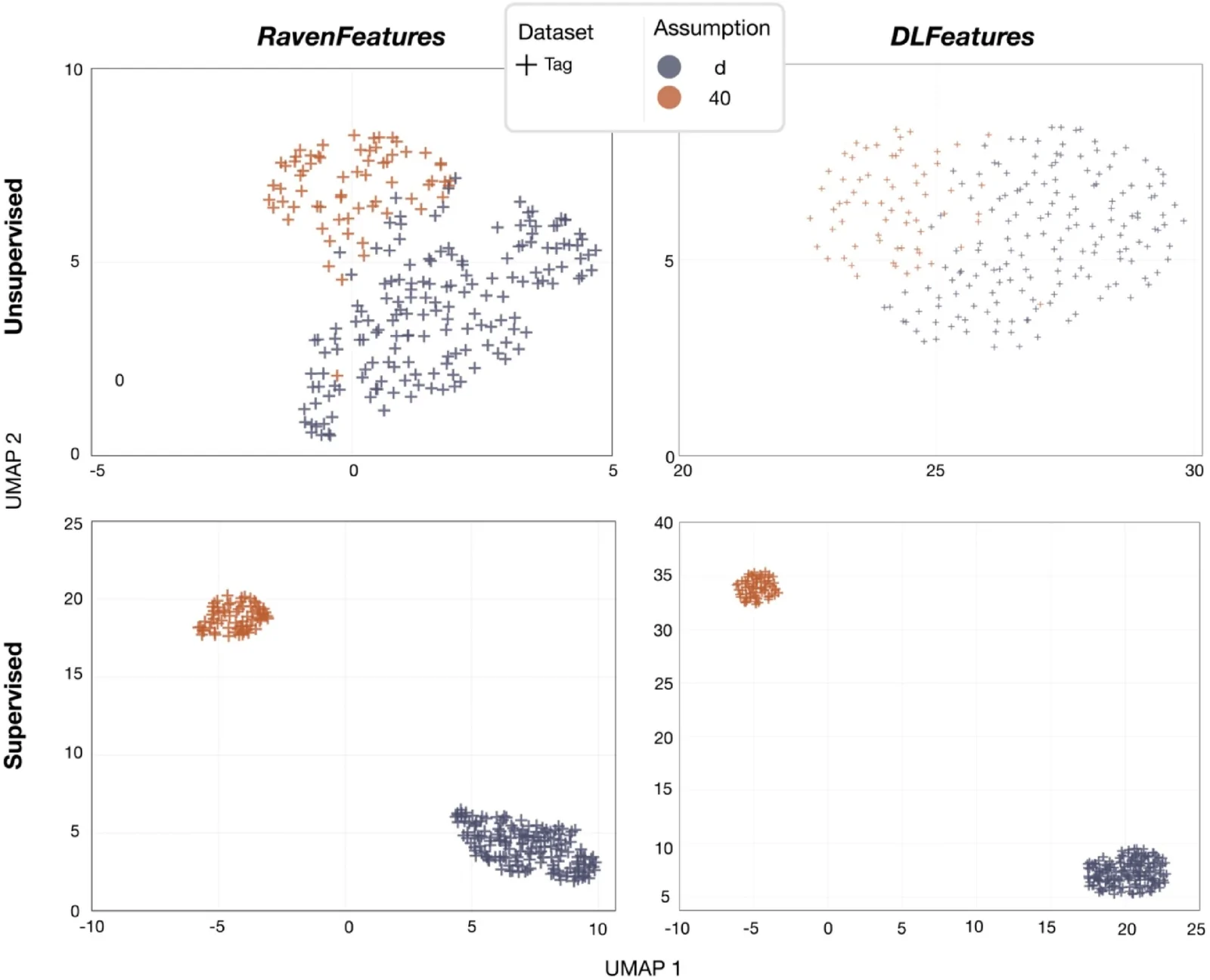
Dimension reduction using UMAP on Tag-RavenFeatures and Tag-DLFeatures. Panels show UMAP projections generated using unsupervised, and supervised approaches. 40 Hz-calls of fin whales (40 - orange), and D-calls of blue whales (*d* - blue) from the tag data are shown accordingly.

As clustering analyses of the moored PAM data alone do not provide a clear basis for reliable attribution of calls to blue whales or fin whales (i.e., in form of two clearly separated clusters with almost all datapoints assigned to them), UMAP projections were generated for the ground-truth Tag dataset to examine cluster separation between call types and to assess whether this dataset could serve as a reference for mooring data. For both feature types (Raven and deep learning), UMAP projections were generated under unsupervised and supervised conditions (Fig. 6). The unsupervised conditions are comparable to the approach presented above for the mooring data, whereas the supervised approach is a technique that incorporates label information to guide the structure of the projection. Under unsupervised conditions, the Tag-RavenFeature projections show a single broad cluster, with clear areas of points sharing the same label but still with substantial overlap between call types. Similar patterns were observed for the DLFeature representations, which likewise formed broad clusters with local label-specific groupings. Under supervised conditions, both Raven- and DLFeature projections exhibited two visually distinct and separated clusters corresponding to D-calls and 40 Hz-calls. As supervised UMAP incorporates class labels during dimension reduction, the separation observed here primarily reflects this constraint.

For consistency with the analyses performed on the Mooring-datasets, HDBSCAN was applied to the unsupervised and supervised UMAP projections of both the Tag-RavenFeatures and Tag-DLFeatures datasets. Table 3 summarizes the results for the HDBSCAN on RavenFeatures and the DLFeatures for the Tag data. While in the unsupervised mode RavenFeatures assigned a larger proportion of calls to clusters, the DLFeatures achieved a slightly higher overall cluster purity, although substantially more calls were classified as noise. When HDBSCAN was applied to the supervised UMAP projections, both the Raven- and DLFeatures achieved perfect agreement with the known species labels.

Clustering performance is further evaluated using four extrinsic clustering metrics based on the known species labels of the Tag dataset. These extrinsic metrics assess how well the clustering assignments correspond to the known ground-truth labels of D-calls and 40 Hz-calls. The resulting metric values indicate correspondence between HDBSCAN-derived clusters and species labels for both feature representations, although agreement is consistently higher for the RavenFeatures than for the DLFeatures-GoogleWhale. The DLFeatures achieve an Adjusted Rand Index of 0.555, compared to 0.778 for the RavenFeatures (Table 4). Similarly, Normalized Mutual Information and V-measure decrease from 0.688 for the RavenFeatures to 0.414 for the DLFeatures. Fowlkes-Mallows scores are high for both feature representations (0.906 and 0.868, respectively). For the HDBSCAN on the supervised projection, both the RavenFeatures and DLFeatures achieved perfect agreement with the known species labels, with all extrinsic clustering metrics yielding 1.0.

**Table 3:** Number and proportion of data points per cluster based on the two call types − 40 Hz-calls of fin whales (40), and D-calls of blue whales (d) of the Tag-RavenFeatures, and the corresponding Tag-DLFeatures-GoogleWhale as determined by the HDBSCAN algorithm. Each entry shows the count of data points belonging to a given call type within a cluster, followed by the corresponding percentage (in parentheses) relative to the total number of data points in the respective cluster. Data points considered as noise by the algorithm are listed as unassigned in the cluster column.

Projection type	Dataset	Cluster	No. of ‘40’ (%)	No. of ‘d’ (%)	No. total
Unsupervised	Tag- RavenFeatures	Unassigned	2 (40%)	3 (60%)	5
		1	63 (84%)	12 (16%)	75
		2	1 (0.7%)	150 (99.3%)	151
	Tag- DLFeatures- GoogleWhale	Unassigned	32 (39.0%)	46 (61.0%)	78
		1	20 (90.9%)	2 (9.1%)	22
		2	14 (10.6%)	117 (89.3%)	131
Supervised	**Tag-RavenFeatures**	**Unassigned**	**0 (0%)**	**0 (0%)**	**0**
		**1**	**66 (100%)**	**0 (0%)**	**66**
		**2**	**0 (0%)**	**165 (100%)**	**165**
	**Tag-DLFeatures-GoogleWhale**	**Unassigned**	**0 (0%)**	**0 (0%)**	**0**
		**1**	**66 (100%)**	**0 (0%)**	**66**
		**2**	**0 (0%)**	**165 (100%)**	**165**

**Table 4:** Extrinsic evaluation of clustering structure in the ground-truth Tag dataset for both RavenFeatures and DLFeatures. Performance is assessed using the Adjusted Rand Index (ARI), Normalized Mutual Information (NMI), V-measure, and Fowlkes–Mallows score. These metrics quantify the degree of agreement between HDBSCAN clustering assignments and known call types (D- and 40 Hz-calls), capturing different aspects of label-cluster correspondence including pairwise agreement, information overlap, and harmonic mean relationships between precision and recall.

Projection type	Dataset	Adjusted Rand Index (ARI)	Normalized Mutual Information (NMI)	V-measure	Fowlkes-Mallows
Unsupervised	Tag- RavenFeatures	0.778	0.688	0.688	0.906
	Tag- DLFeatures- GoogleWhale	0.555	0.414	0.414	0.868
Supervised	Tag- RavenFeatures	1.0	1.0	1.0	1.0
	Tag- DLFeatures- GoogleWhale	1.0	1.0	1.0	1.0

#### Tag dataset as reference for FM call attribution

To assess whether the Tag dataset can serve as a reference for attributing call types in passive acoustic monitoring data, two approaches were evaluated partially excluding some of the tag data to serve as an evaluation set. For each species, one deployment was randomly excluded from the Tag dataset prior to building the embedding, representing 12% of all available fin whale calls and 15% of all available blue whale calls (spanning a Duration 90% range of 0.4–1.2 s).

**Fig. 7:**
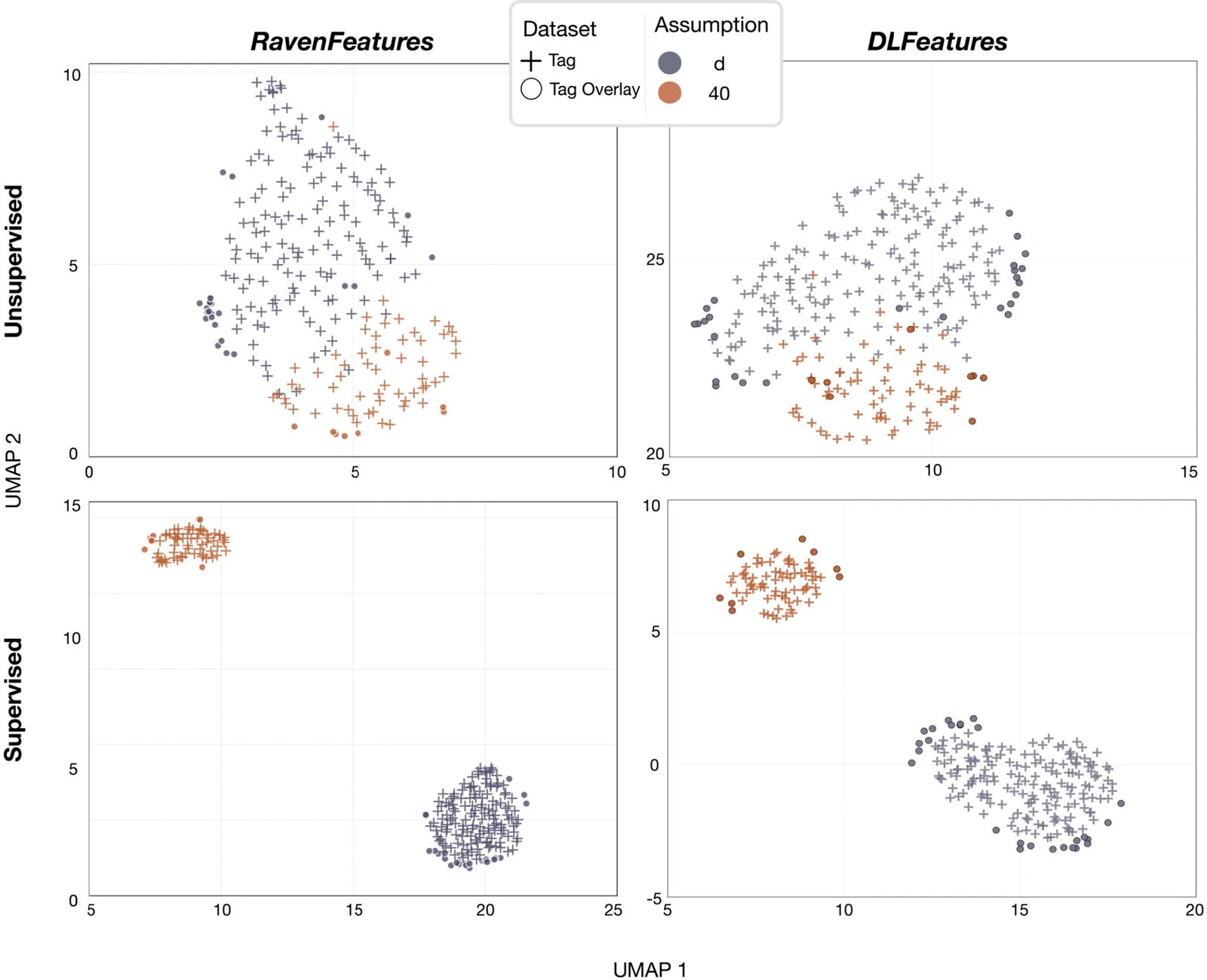
UMAP projections of Tag-RavenFeatures and Tag-DLFeatures (GoogleWhale) under leave-one-deployment-out validation. For each species, one deployment was excluded from UMAP construction and subsequently projected into the embedding generated from the remaining Tag data. Panels show unsupervised and supervised embeddings. Crosses indicate calls used to construct the embedding, whereas circles (“Tag Overlay”) indicate calls from the excluded deployments projected into the UMAP space. Fin whale 40 Hz-calls are shown in orange (40) and blue whale D-calls in blue (*d*).

The first approach was to generate UMAP projections using the training Tag data, and calls from the excluded deployments were projected into the resulting embedding space, hereinafter referred to as Tag Overlay (Fig. 7). This means that the feature vectors of the excluded calls were transformed into the same two-dimensional projection (dimensionality-reduced representation) without using label information, allowing evaluation of whether calls from excluded deployments aligned with the species-specific patterns observed in the remaining Tag data.

For both RavenFeatures and DLFeatures, the resulting UMAP projections showed patterns comparable to those observed when all Tag data were included (comparison with Fig. 6). Under unsupervised conditions, the Tag Overlay aligns with the local species-specific groupings within the RavenFeature embedding, with a similar but more structured pattern observed for the DLFeatures. Under supervised conditions, both feature representations show clear separation into two clusters, with the Tag Overlay aligning consistently with the corresponding species-specific clusters.

The second approach was to train a Random Forest model. The classifier was trained using the Tag-dataset excluding the left-out deployments and evaluated using the Tag Overlay. For both the RavenFeatures and the DLFeatures, the Random Forest classifier correctly classified all calls from the excluded deployments, achieving a classification performance of 1.0 on the left-out data. Feature importance analysis on the RavenFeature classification indicates that duration-related parameters contributed most strongly to classification, with Duration 90% and Delta Time showing the highest importance. This suggests that call duration remains the strongest parameter for distinguishing between blue and fin whale FM calls, while additional acoustic features also contribute to classification.

#### Applying tag-trained approaches to moored PAM data

Finally the tested approaches should be applied to assign a species-identity to the FM calls of unknown source from the Mooring-dataset. The Mooring-dataset was therefore projected into the UMAP projection space constructed from the Tag-RavenFeatures and Tag-DLFeatures, allowing visual comparison of call distributions and assessment of similarities between the known (tagged) calls and the unassigned FM calls from the mooring data.

**Fig. 8:**
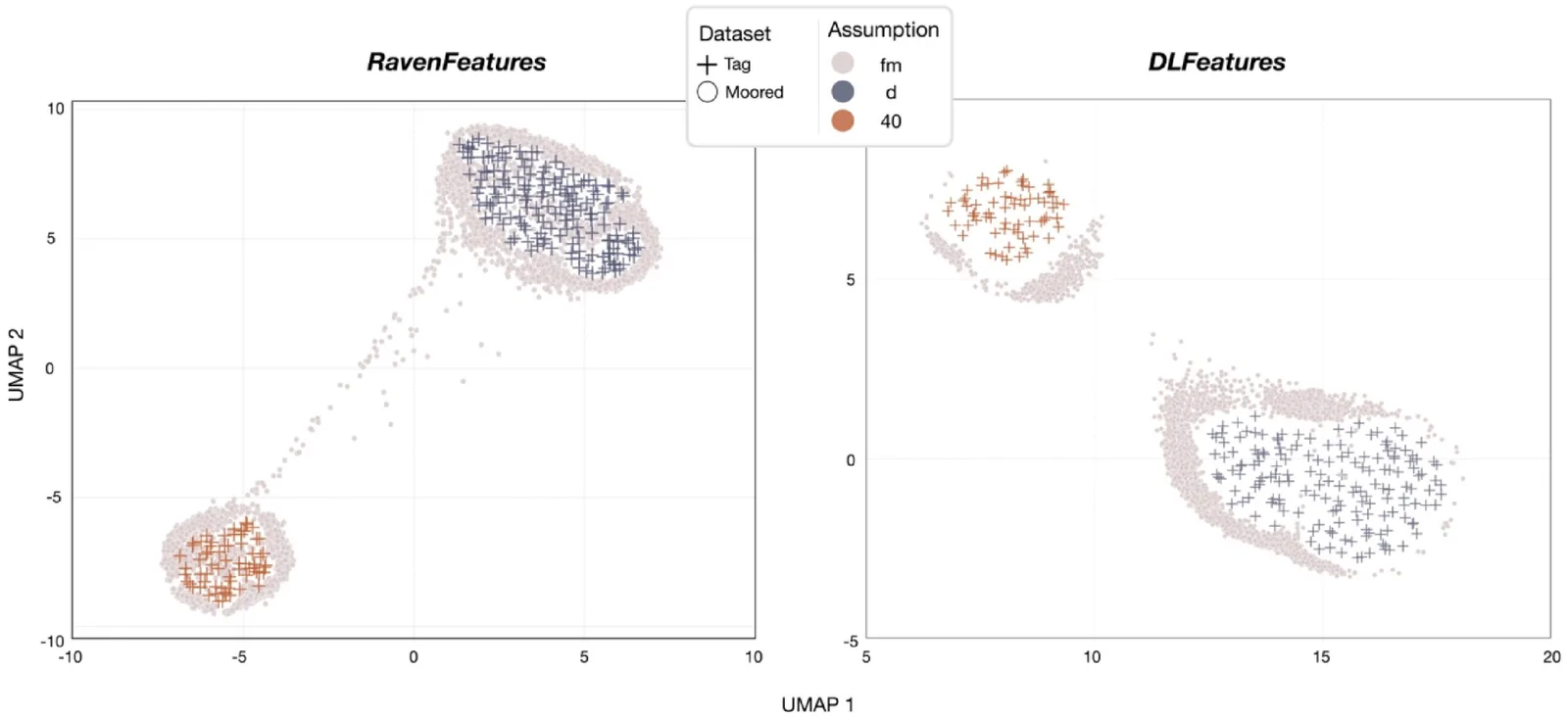
Dimension reduction using UMAP on Tag-RavenFeatures and Tag-DLFeatures-GoogleWhale. Panels show UMAP projections generated using a supervised approach. The Mooring-RavenFeatures and -DLFeatures (GoogleWhale), are projected onto the respective Tag-UMAP spaces and their source is assumed unknown (fm - light grey). 40 Hz-calls of fin whales (40 - orange), and D-calls of blue whales (*d* - blue) from the tag data are shown accordingly.

UMAP projections of Tag-RavenFeatures and Tag-DLFeatures were generated under supervised conditions (Fig. 8), as under unsupervised conditions no clear cluster separation could be achieved. While a substantial proportion of the Mooring-RavenFeatures overlay aligns with the two distinct clusters corresponding to D-calls and 40 Hz-calls formed by the Tag-RavenFeatures, other points form a continuous distribution between these. In contrast, the supervised projections of the DLFeatures shown in Fig. 8 produced two distinct clusters based on class labels, with the Mooring-DLFeatures data aligning strongly with the two clusters.

To be able to use the UMAP projection strategy for classification, species attribution was performed using a five-nearest-neighbour approach in the UMAP space. Only 214 calls (2.5%) remained unassigned for the RavenFeatures, compared with just five calls (0.1%) for the DLFeatures. In the RavenFeature projection, unassigned calls were primarily located within the transition region connecting the two species-specific clusters, whereas attributed calls closely followed the corresponding tagged call distributions. In the GoogleWhale DLFeature projection, the five unassigned calls also occurred between the two species-specific clusters, but all in close proximity to the blue whale cluster.

**Fig. 9:**
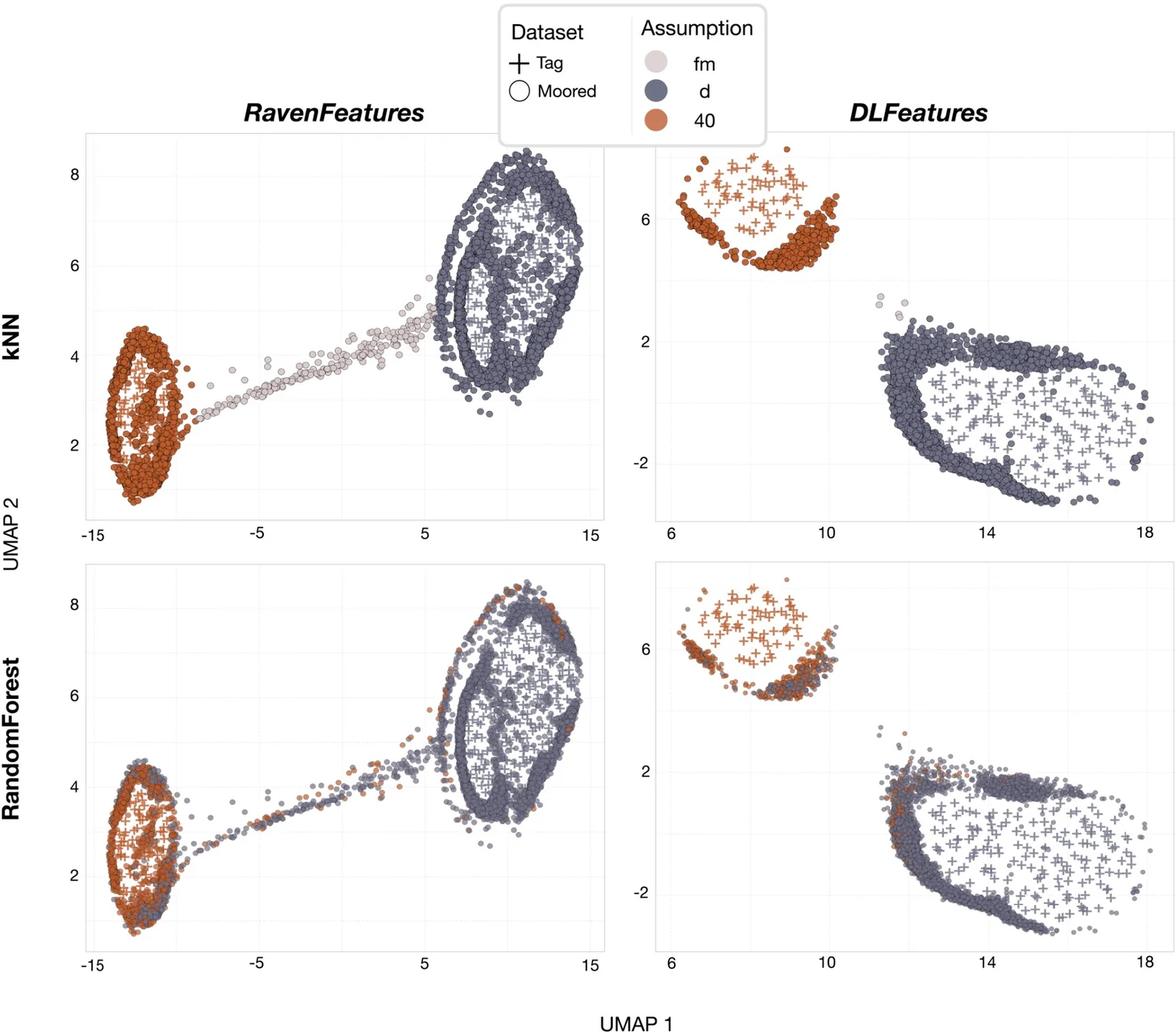
Dimension reduction using supervised UMAP on the Tag-RavenFeatures and Tag-DLFeatures (GoogleWhale). The Mooring-RavenFeatures and Mooring-DLFeatures are projected onto the corresponding Tag-UMAP projection spaces. Mooring calls are coloured according to the species attribution obtained using the k-nearest-neighbour (kNN) approach (top panels) and the Random Forest classifier (bottom panels). Fin whale 40 Hz-calls are shown in orange (40), blue whale D-calls in blue (*d*), and remaining unknown calls (fm) in grey.

Also Random Forest classifiers trained on the complete Tag dataset were subsequently applied to the Mooring-dataset to predict species identity. The resulting classifications are visualized in Fig. 9 by coloring the Mooring overlay according to the Random Forest predictions. Overall, the predicted labels broadly follow the species-specific cluster structure observed in the supervised UMAP projection. However, local regions of intermixed fin and blue whale classifications are evident, particularly near the boundaries between clusters, indicating that the Random Forest predictions do not fully coincide with the UMAP-based neighbourhood attribution. Species attributions obtained using the supervised UMAP-kNN approach and Random Forest classifiers are summarized in Table 5.

**Table 5:** Species attribution of FM calls in the Mooring-datasets obtained using the supervised UMAP-kNN and RandomForest approaches based on RavenFeatures and GoogleWhale-DLFeatures. Values indicate the number and percentage assigned to fin whale 40 Hz-calls, blue whale D-calls, or remaining unassigned. Species identity of the Mooring calls is unknown; therefore, these values represent predicted species attributions rather than classification accuracy.

Classification approach	Dataset	40 Hz	D-call	Not attributed
UMAP + kNN	RavenFeatures	2725 (31.9%)	5598 (65.6%)	214 (2.5%)
	DLFeatures- GoogleWhale	625 (7.4%)	7723 (92.5%)	5 (0.1%)
RandomForest	RavenFeatures	2015 (23.6%)	6522 (76.4%)	0 (0%)
	DLFeatures- GoogleWhale	916 (10.9%)	7437 (89.1%)	0 (0%)

## Discussion

We investigated acoustic differences between blue and fin whale FM calls, D-calls and 40 Hz-calls, using long-term PAM and tag recordings with the goal of identifying acoustic differentiation rules for distinguishing between the two species. FM calls could not be reliably separated by models using long-term PAM data alone, whereas tag recordings with confirmed species identity revealed approaches that allow distinction. The described approach supports limited but potentially meaningful attribution of FM calls to species in datasets where species identity is otherwise unknown (i.e., moored, long-term PAM datasets).

Important limitations must be considered when interpreting our results. One limitation arises from analyst bias during manual detection and boxing of calls in Raven^32^. Call parameters are derived from the selection box drawn around the call. This process is sensitive to spectrogram settings such as time-frequency resolution and the color scale chosen^33^, with the potential to introduce systematic differences across datasets. The application of deep learning features is robust to this type of measurement-error, however, a soundscape bias (i.e., related to the geographic location) can in turn affect this method of differentiation. An additional limitation is that tag recordings of blue and fin whales represent different populations from those occurring in Antarctic waters. Although 40 Hz-calls and D-calls have been described across many populations globally [e.g., ^12,13,33–35^], little population-specific differences may influence the results. Thus, the results presented in this study should be interpreted accordingly.

The distinction of FM calls in long-term PAM datasets has to date primarily relied on differences in acoustic parameters. Call duration (Delta Time), has been proposed as the key parameter for distinguishing between blue and fin whale FM calls in previous studies^15,24^. Our examination of the ground-truth tag recordings, where species identity is confirmed, showed that this parameter does not reliably separate 40 Hz-calls and D-calls. Most acoustic parameters overlapped substantially between species, including call duration (Delta Time), with 98.5% of 40 Hz-calls falling within the D-call range. Calls longer than ~ 1.5 s were D-calls only, suggesting that this threshold could serve as a simple D-call classification criterion if classification of all calls is not essential. In contrast, Duration 90% (Dur90%), a robust measurement of call duration defined as the interval between the 5% and 95% cumulative energy points^33^, provided the greatest separation between species. In the tag dataset, fin whale FM calls reached a maximum Duration 90% of 0.612 s, whereas blue whale FM calls had a minimum of 0.612 s, indicating minimal overlap and suggesting a potential boundary. Although considering that transmission loss is expected to affect this parameter^36^, it is less sensitive to analyst bias than other measures. To ensure confident parameter-based classification in long-term PAM datasets, analysis could be restricted to high-quality calls with SNR NIST Quick (dB) > 12 dB (the minimum SNR NIST Quick (dB) value in the filtered Mooring-dataset). FM calls could then be assigned to fin whales if Duration 90% < 0.6 s, and to blue whales if Duration 90% > 0.7 s. Yet, while these proposed thresholds may provide a parameter-based separation for FM calls in long-term PAM datasets, they inevitably exclude intermediate calls and lower-quality signals, and should be applied with caution.

In addition to acoustic parameters, previous studies have proposed contextual labelling as an alternative approach for species attribution in long-term PAM datasets. Applying this approach to the Mooring-dataset did not reproduce the acoustic separation observed in the ground-truth Tag dataset. Instead, substantial overlap remained across all measured Raven features, including Duration 90%, which provided the clearest distinction between species in the Tag recordings. Contextual labelling also produced different species attributions than the ground-truth-derived Duration 90% rule, with only approximately 45% agreement for calls attributed as fin whale 40 Hz-calls or blue whale D-calls. One possible explanation is that acoustic context does not necessarily reflect the identity of the individual producing the FM call. For example, an FM call produced by one species may occur while only the stereotyped call type of the other species is detected within the surrounding time window, resulting in incorrect attribution. Together, the above findings suggest that acoustic context alone does not provide a reliable method for species attribution and that, despite its limitations, the Duration 90% approach currently provides a more robust basis for distinguishing between blue and fin whale FM calls in long-term PAM datasets.

Beyond the exploration of individual acoustic parameters, clustering analyses were used to assess whether FM calls exhibit groupings in acoustic feature space. Unsupervised UMAP projections of the Mooring dataset did not reveal distinct clusters. Instead, both RavenFeatures and DLFeatures formed largely continuous distributions, although the DLFeature UMAP space shows a slightly more structured distribution with several locally denser regions. Similarly, HDBSCAN identified only a small number of broad clusters together with a substantial proportion of unassigned calls, suggesting that the Mooring-dataset does not contain clearly separable acoustic groups in the absence of ground-truth information. Although intrinsic clustering metrics indicated more compact and better separated clusters for the DLFeatures than for the RavenFeatures, these differences were insufficient to resolve distinct species-specific call groups. In contrast, UMAP projections of the Tag-RavenFeatures and DLFeatures revealed more pronounced separation between fin and blue whale calls. Under unsupervised conditions, some overlap remained between call types, indicating that complete separation of FM calls remains challenging even in recordings with confirmed species identity and minimal transmission loss effects. Nevertheless, the DLFeatures consistently exhibited overall clearer separation than the RavenFeatures. This pattern was reflected by the HDBSCAN results, where the DLFeatures formed cleaner clusters, whereas the RavenFeatures produced clusters with greater species overlap, although assigning more calls to clusters. Although the extrinsic clustering metrics were consistently lower for the DLFeatures, it should be noted that these metrics were calculated only for calls assigned to HDBSCAN clusters and therefore exclude calls classified as noise. Consequently, they primarily reflect agreement among clustered calls and should be interpreted together with the proportion of unassigned calls in the DLFeature projection. In contrast, HDBSCAN applied to the supervised UMAP projections achieved perfect agreement with the known species labels for both feature representations.

The leave-one-deployment-out analysis further demonstrated that previously unseen tag recordings were consistently projected into the corresponding species-specific clusters of the supervised UMAP spaces. The Tag dataset therefore seems to provide a suitable reference space for the projection of previously unseen FM calls. Random Forest classification trained on Tag data was additionally assessed as a means of assigning call types on mooring data, identifying Duration 90% as the most important predictor for the RavenFeatures, which further supports its importance for distinguishing between fin and blue whale FM calls. The Random Forest also incorporated additional acoustic features into the classification, indicating that additional information beyond a single duration-based parameter contributes to the differentiation of fin and blue whale FM calls. These findings further support the use of multivariate feature representations for projection-based species attribution, particularly deep learning feature embeddings, which appear to capture acoustic characteristics that cannot readily be described by individually extracted Raven features.

While performance on the left-out tag data was perfect, when applied to the moored data the predicted classes were not always close to the obtained clusters using Tag data, suggesting that the learned differentiation strategy by the RandomForest was not representative of real-case moored data. Direct comparison with previously proposed separation approaches is limited, such as Ou et al.^10^ and Rasmussen & Širović^37^, as in all cases species-identity from long-term mooring data is a questionable ground-truth when both species theoretically occur in the recording area. While Ou et al.^10^, for example, trained a support vector machine classifier on manually extracted pseudo Wigner-Ville features using species labels derived from long-term passive acoustic recordings, the present study used independently confirmed species identity from animal-borne tag recordings. As the replication of non-standardized manual measurements is not robust enough to ensure comparability, we would instead propose that future studies could try the pseudo Wigner-Ville distribution visualization as the input for deep learning feature extraction to comparably evaluate its potential for attributing FM calls to species.

Given the lack of long-term, ground-truthed moored PAM data for blue and fin whales, and the demonstration that a model trained on the small Tag dataset might not generalize well when applied to moored data, we propose a framework which combines supervised, semi-supervised and unsupervised methods. Projecting the Mooring dataset into the Tag UMAP space demonstrated potential for species attribution of previously unseen FM calls. In the RavenFeature UMAP, a large proportion of mooring calls are aligned with the corresponding tagged call clusters, while other points form a continuous distribution between these two clusters. In contrast, in the DLFeature space consistently all mooring calls are projected directly into one of the two tag-derived clusters, suggesting a clearer separation of call types when multiple learned acoustic features are considered simultaneously. Using the k-nearest-neighbour (kNN) approach, species attributions could be assigned directly within the UMAP space while simultaneously evaluating the position of each attributed and unattributed call relative to the ground-truth Tag recordings. In the RavenFeature space, calls that remained unassigned were primarily located within the transition region between the two tagged species clusters. In contrast, almost all mooring calls projected directly into one of the two DLFeature clusters, resulting in only five unattributed calls.

While the Random Forest classified every call into one of the two species, the projection-based approach retained the possibility of leaving acoustically ambiguous calls unassigned, e.g., the continuous connection of calls between the tag-derived clusters in the RavenFeature UMAP. As the RandomForest identified Duration 90% as the most important predictor for the RavenFeatures, its reliance on this parameter may contribute to some of the differences observed between the RandomForest classifications and the projection-based kNN attribution, since the distribution of Duration 90% across the projections (see Appendix Fig. A4) does not fully correspond to the species projection structure. In contrast, the UMAP projection allows each attribution to be interpreted in the context of its acoustic proximity to known tagged calls. This is particularly the case in the DLFeature UMAP, where the well-defined tag-derived clusters allow projected mooring calls to be interpreted directly relative to the ground-truth reference. We therefore consider a more conservative approach, in which uncertain calls remain unassigned, preferable to maximizing the number of attributed calls at the expense of potentially incorrect species assignments. However, the increased separability and clearer clusters obtained with our proposed framework with respect to training a simple supervised model on the Tag Data alone, relies on the assumption that calls from the two species are acoustically distinct and can be separated, while calls within each species remain sufficiently similar. Only access to ground-truthed, long-term moored PAM data can be used to fully confirm this hypothesis.

Overall, this study refines the previously proposed duration-based approach while introducing a complementary framework for attributing blue and fin whale FM calls. Both approaches are not context-based and therefore not spatially or temporally restricted. The Duration 90% thresholds, Duration 90% < 0.6 s for fin whales, and Duration 90% > 0.7 s for blue whales presented here provide a more robust alternative to the previously used call duration criterion for high-quality recordings (SNR NIST Quick (dB) > 12 dB), allowing conservative parameter-based differentiation of FM calls. This may be useful for manual inspection of spectrograms, however, manual classification requires expertise and is prone to observer bias^32^, limiting its reliability. The main advance of this study is the integration of animal-borne tag recordings with confirmed species identity to inform species attribution of FM calls in long-term passive acoustic monitoring datasets. The species-confirmed tag recordings enabled both the refinement of the Duration 90% criterion and the construction of the supervised reference space. Without this ground-truth reference, neither approach would have been possible. Rather than relying on a single acoustic parameter or a conventional classifier, the proposed projection-based framework projects deep learning features of previously unseen FM calls into this supervised reference space, combining information from multiple acoustic features while allowing unknown calls to be interpreted directly relative to the ground-truth reference. This allows species attribution to be interpreted in the context of acoustic similarity and provides an explicit measure of uncertainty by leaving acoustically ambiguous calls unassigned. Both methods are restricted to high-quality calls (SNR NIST Quick (dB) > 12), which substantially limits the proportion of FM calls for which species attribution is currently feasible. Lowering the SNR NIST Quick (dB) threshold may eventually be possible if long-term PAM recordings become available that can be paired with simultaneous sightings confirming the exclusive presence of a single species. Yet this remains challenging given the broad distributional overlap of blue and fin whales and the transmission distance of their FM calls of up to 18 km^13,18,19,38^.

Deep learning features have proven effective for call classification^39,40^. However, although the projection-based approach presented here appears to assign FM calls consistently, the deep learning features extracted by the pre-trained models are computed from the full frequency range of each audio snippet, whereas the Raven features are extracted only from the manually annotated frequency limits of the FM call. Consequently, the extracted feature representations may be influenced not only by the call itself but also by the surrounding soundscape, including biotic, abiotic and anthropogenic background noise^40–43^. As a result, part of the observed separation may reflect recording-site characteristics in addition to species-specific call properties. Our current approach does not allow identification of which aspects of the audio snippet contribute most strongly to the learned deep learning representation. Explainable artificial intelligence methods, such as layer-wise relevance propagation^44^ or DeconvNet^45^, represent promising approaches for interpreting deep learning embeddings, but were beyond the scope of this study. Future work combining such approaches with expanded ground-truth tag datasets could help to identify the characteristics underlying the observed differences between the RavenFeature- and DLFeature-based species attributions, including the differing numbers of attributed and unassigned calls. This will be important for evaluating which of the two proposed approaches provides the more reliable basis for species attribution. In addition to the methodological considerations, the applicability of the proposed framework is constrained by the availability of suitable ground-truth reference data. The Tag recordings used in this study originate from blue whales off California and fin whales off Chile, whereas the Mooring-dataset represents Antarctic populations. Although FM calls have been described across populations worldwide and are not generally considered to exhibit pronounced population-specific differences (e.g.^10,12,13,15,21,34^), population- or hemisphere-specific variation cannot be excluded. Ideally, animal-borne tag recordings would be collected simultaneously with passive acoustic monitoring data from the same region and acoustic environment. Such data would provide the most representative reference for species attribution while minimizing both potential soundscape bias and uncertainty associated with population differences. However, to our knowledge, acoustic tag recordings are currently unavailable for Antarctic blue and fin whales. Despite the population and soundscape mismatch, the Mooring calls projected closely around the ground-truth Tag recordings, indicating that the proposed framework captures species-related acoustic characteristics across datasets. Although, the relative influence of population-specific call characteristics and recording-site soundscapes on the learned embeddings cannot currently be resolved. Consequently, species attributions presented here should be interpreted with appropriate caution. Expanding ground-truth tag datasets across multiple populations and acoustic environments will allow increasingly representative UMAP reference spaces to be created, improving both the robustness and the broader applicability of projection-based species attribution.

Together, the classification approaches presented here provide a practical framework for classifying blue and fin whale FM calls in long-term PAM datasets. Integrating independently confirmed tag recordings with passive acoustic monitoring data extends species attribution beyond parameter-based classification alone and provides an interpretable reference framework for previously unseen FM calls. Applying these methods can expand knowledge of species occurrence during non-song periods, inform on acoustic presence and feeding behavior^13,46^, and support effective monitoring and management efforts. While currently restricted to high SNR calls, this study demonstrates that integrating ground-truth tag data into PAM analyses is a promising avenue for improving species attribution and monitoring efforts.

## Methods

### Data acquisition and labelling

#### Mooring data

**Fig. 10:**
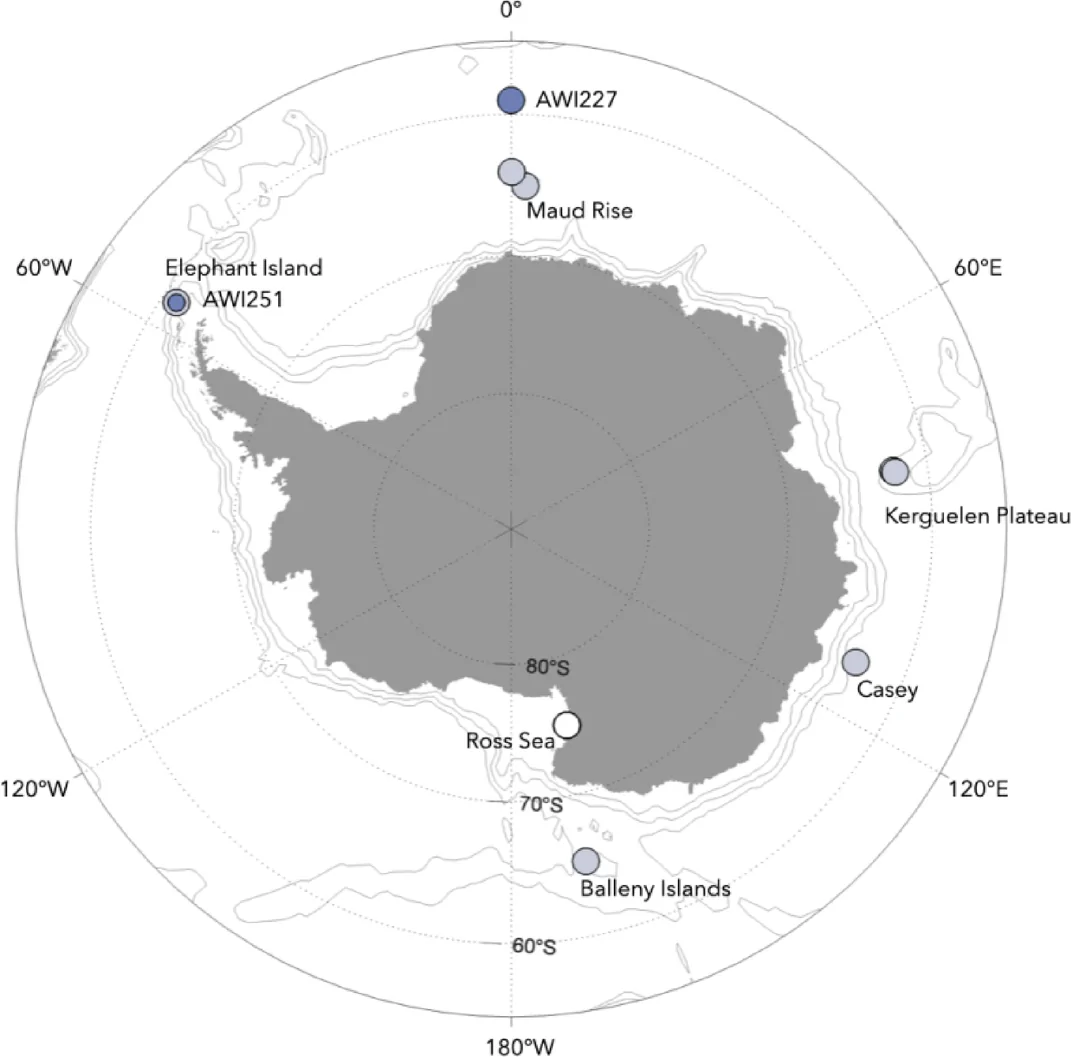
Map of the Southern Ocean showing the locations of passive acoustic recorders contributing to the datasets used in this study. AWI recorder sites (AWI251 and AWI227) are indicated in blue. Six additional sites from the open-access dataset where FM calls were detected are shown in lightgrey, while the Ross Sea mooring, where no FM call data were detected, is shown in white. Note that the Elephant Island recording site from the open-access dataset and AWI251 are overlapping in the map due to their nearly identical coordinates. The map was generated using M_Map^47^ in MATLAB (Version 2022b; The MathWorks Inc., 2022).

Manually annotated FM calls of fin and blue whales were compiled from multiple long-term mooring-mounted hydrophone datasets in the Southern Ocean (see Fig. 10; Table 6 for details). One primary source was recordings from the Alfred Wegener Institute (AWI), collected at two sites in the Weddell Sea: AWI251 off Elephant Island and AWI227 on the Greenwich Meridian. An additional source was an open-access annotated library of underwater acoustic recordings, developed for testing and training automated detection algorithms for Antarctic blue and fin whale sounds^15^. This dataset includes recordings from seven sites across the Southern Ocean, spanning five years (2005, 2013–2015, 2017), with site coverage varying by year.

**Table 6:** Summary of annotated passive acoustic mooring datasets used in this study. This table includes both manually annotated datasets (AWI) and datasets obtained from the open-access library of underwater acoustic recordings^15^. Available call types include fin whale 20-Hz calls (20), blue whale A-, B-, and Z-calls (abz), as well as frequency-modulated (FM) calls from either species.

Data source	Dataset ID	Latitude	Longitude	Analysed period	Total hours annotated (h)	Available call types	Total no. fm calls	Data access
AWI	AWI227	59 2.82° S	000 5.78° E	2012-12-11–2013-07-11	140	FM, abz, 20	1507	https://doi.org/10.1594/PANGAEA.966612
	AWI251	61 0.13° S	55 97.78° W	2013-01-18–2013-12-31	86.6	fm	528	https://doi.org/10.1594/PANGAEA.966766
Open-access data[Miller et al. 2021^23^]	Open access dataset from 7 Southern Ocean sites over 5 years (2005, 2013–2015, 2017); sites not consistently represented across years.				1661	FM, abz, 20	16174	doi:https://doi.org/10.26179/5e6056035c01b

For conformity between the open access and AWI data, AWI recordings were decimated to a sampling rate of 250 Hz and visually inspected for blue and fin whale calls using the sound analysis software Raven Pro 1.6 (The Cornell Lab of Ornithology, Center for Conservation Bioacoustics, Ithaca, NY). Analysis was performed using smoothed spectrograms with a Hanning window, with window and DFT size of 256 and 80% overlap. For the AWI227 dataset annotations included FM calls, clearly identifiable fin whale 20-Hz pulses, and blue whale A-, B-, and Z-calls. In contrast, the AWI251 dataset was manually analyzed for FM calls only. Calls were manually identified and annotated based on spectrogram visual inspection. Call onset and offset were defined as the visually detectable beginning and end of the frequency-modulated downsweep, corresponding to the period of sustained signal energy above background noise. This annotation procedure was applied consistently across all datasets. For each annotation, 12 quantitative parameters describing call characteristics were extracted from Raven Pro (see Table 7) for both AWI and open-access datasets. The two datasets were then combined into a single dataset. To improve data quality and ensure that only the most representative calls were included, a ‘Mooring-dataset’ subset was created by excluding all FM calls with a signal-to-noise ratio (SNR) proxy below 12 dB (SNR NIST Quick (dB) > 12). This threshold was chosen to minimize the influence of low-SNR detections and emphasize well-defined, high-quality acoustic signals.

**Table 7:** Raven features to describe detected FM calls of fin and blue whales in the various datasets. Parameters were calculated according to the temporal and spectral limits of the respective vocalizations by drawing selection boxes around detected vocalizations. Details on measurements according to the Raven Pro 1.4 User’s Manual^33,48^.

Measurement	Description
Low frequency	Lower frequency limit of the selection box in Hz.
High frequency	Upper frequency limit of the selection box in Hz.
Delta frequency	The difference between the upper and lower frequency limits of the selection box in Hz.
Center frequency	The frequency that divides the selection into two frequency intervals of equal energy in Hz.
Peak frequency	The frequency at which the maximum amplitude occurs within the selection in Hz.
Frequency 25%	The frequency that divides the selection into two frequency intervals containing 25% and 75% of the energy in Hz.
Frequency 75%	The frequency that divides the selection into two frequency intervals containing 75% and 25% of the energy in Hz.
Frequency 5%	The frequency that divides the selection into two frequency intervals containing 5% and 95% of the energy in Hz.
Frequency 95%	The frequency that divides the selection into two frequency intervals containing 95% and 5% of the energy in Hz.
Delta Time	The difference between begin time and end time of the selection in s.
Duration 90%	The difference between the point in time that divides the selection into two time intervals containing 5% and 95% of the energy (Time 5%) and the point in time that divides the selection into two time intervals containing 95% and 5% of the energy in the selection in s.
Slope	The slope of the selection, calculated as delta frequency divided by delta time in Hz/s.
SNR NIST Quick	Signal-to-noise ratio (SNR) estimated as the difference between root mean square (RMS) power values that divide the selection into 15% (noise level) and 85% (signal level) of windows in dB, using overlapping 20 ms windows. This feature provides an approximate measure of the selection’s signal strength relative to ambient noise.

#### Blue and fin whale acoustic tag data

An acoustic tag dataset (Tag-dataset) was included, consisting of FM calls recorded during acoustic tag deployments on blue and fin whales, providing species-verified labels. This dataset comprised 165 blue whale D-calls recorded during three deployments off the coast of California, as well as 66 fin whale 40 Hz-calls documented across 12 deployments off Chile (see Table 8 for details).

All tag recordings were decimated to 250 Hz and manually analyzed in Raven Pro 1.6 using the same spectrogram settings and measurements as those applied to the Mooring-dataset. Because the calls were recorded from individually tagged animals, species identity is confirmed. Accordingly, labels in this dataset are treated as ground truth, and no unassigned FM calls are present.

**Table 8:** Summary of the tag datasets used as ground-truth in this study. This table includes the manually annotated tag datasets on blue and fin whale FM calls, D- (d) and 40 Hz- (40) calls respectively, from deployments off California and Chile.

Data source	Species tagged	Latitude	Longitude	No of deployments	Total hours analysed (h)	Call type	Total no. calls	Data availability
Isla Chañaral, Chile	fin whale	29 3.53° S	71 57.94° E	12	~ 333.4	40	66	Buchan, Unpublished
California, USA	blue whale	33 47.96° N 33 42.85° N	118 31.09° E 118 23.87° E	3	~ 44.6	d	165	Lewis 2018^49^

#### Deep learning feature extraction

In addition to the acoustic features extracted using Raven, deep learning features were computed for the audio snippet from the Mooring- and Tag-datasets, referred to as Mooring- and Tag-DLFeatures. These deep learning features are high-dimensional vectors of acoustic properties, representing learned acoustic representations extracted from the final layer of each pre-trained model. Feature extraction was performed using bacpipe^50^, an open-access Python-based software tool. The models selected for evaluation were restricted to those partially or fully trained on underwater or marine PAM data and with a suitable frequency range, namely Biolingual^30^, and GoogleWhale^31^. Biolingual^30^ directly produces a 1D embedding vector due to its transformer-based architecture, which allows variable input window sizes. In contrast, GoogleWhale^31^ operates with fixed input window sizes and produces multi-dimensional embeddings per audio snippet. These multi-dimensional embeddings had to be pooled along the time axis to obtain a 1D vector per audio snippet. We applied median pooling, as it was found to produce more stable representations compared to mean, max, and min pooling. The choice of pooling strategy has been shown to influence embedding structure and clustering behaviour in previous studies^39^.

### Exploratory data analyses and clustering

To assess differences in temporal-spectral parameters among call types, boxplots were used to visualize the distribution of the 12 individual Raven features in the Tag-RavenFeatures dataset. To evaluate a contextual labelling approach, acoustic feature distributions and species attributions obtained for the Mooring-RavenFeatures dataset were compared with those of the Tag-RavenFeatures dataset. A rule-based labelling approach was chosen to assign each FM call in the Mooring-dataset to a presumed species category or to label it as unclassified. FM call labels for the Mooring-dataset were assigned based on the co-occurrence of well-known species-specific vocalisations within a ± 30 min temporal window around each FM call. These included fin whale 20-Hz calls and blue whale A-, B-, and Z-calls. To increase reliability, only high-quality species-specific calls with SNR NIST Quick (dB) > 15 were included. An FM call was assumed to be a 40 Hz fin whale call if only fin whale species-specific calls were present, and a D-call if only blue whale species-specific calls were detected. Calls were labelled as unclassified (FM) if both species were detected, or if no species-specific calls were present within the defined time window.

To further explore potential non-linear structure, a Uniform Manifold Approximation and Projection (UMAP)^51^ was applied to both the Raven and deep learning feature sets to reduce dimensionality and visualize call similarity in a 2D space. UMAP requires specification of two key hyperparameters: the number of neighbors, which balances local and global structure, and the minimum distance, which controls the compactness of the distribution of data points in the reduced space. Because the optimal settings are data-dependent, different ranges were tested for both Raven and deep learning features.

Specifically, n_neighbors ∈ {2, 5, 10, 20, 50, 100} and min_dist ∈ {0.1, 0.3, 0.5} were tested. A configuration of n_neighbors = 5 and min_dist = 0.1 was selected for the Raven features, while n_neighbors = 50 and min_dist = 0.3 was selected for the deep learning features. These settings allowed for a more exploratory view of the projection, revealing structures within broader clusters in the different datasets.

UMAP was applied to the Mooring-dataset in an unsupervised mode, while for the Tag*-* dataset both unsupervised and supervised modes were applied. In the unsupervised setting, the algorithm identifies structure based solely on the input features, without any label information. In contrast, supervised projections fully incorporate label information to guide the structure of the projection.

To quantify potential clustering structure, Hierarchical Density-Based Spatial Clustering of Applications with Noise (HDBSCAN)^48^ was applied to the two-dimensional UMAP projection generated from the Raven feature datasets (Mooring- and Tag-RavenFeatures) and the corresponding deep learning feature datasets, using the hdbscan Python library^48^. The algorithm identifies regions of varying density to form stable clusters, while allowing to consider dissimilar or sparse samples as noise. HDBSCAN parameters were explored individually for each dataset to identify stable clustering solutions while yielding a biologically relevant number of clusters (two to three). For the Tag dataset, clustering was performed using min_cluster_size = 50 and min_samples = 3 for the RavenFeatures, and min_cluster_size = 15 and min_samples = 2 for the DLFeatures. For the Mooring dataset, clustering was performed using min_cluster_size = 500 and min_samples = 10 for the RavenFeatures, and min_cluster_size = 30 and min_samples = 10 for the DLFeatures.

Clustering performance was subsequently evaluated using intrinsic and extrinsic clustering metrics. For the unlabeled Mooring-dataset, intrinsic metrics including the Silhouette Score, Davies–Bouldin Index, and Calinski–Harabasz Index were used to assess cluster compactness and separation^52–54^. The Silhouette Score measures the degree of separation between clusters, with higher values indicating better-defined clusters^52^. The Davies-Bouldin Index quantifies cluster overlap, with lower values indicating better clustering performance^53^. The Calinski-Harabasz Index evaluates the ratio of between-cluster to within-cluster dispersion, with higher values generally indicating stronger cluster structure^54^. For the Tag-dataset, where species labels were available, clustering performance was evaluated using extrinsic metrics including the Adjusted Rand Index (ARI), Normalized Mutual Information (NMI), V-measure, and Fowlkes–Mallows Index by comparing HDBSCAN cluster assignments with the known species labels^55–58^. The Adjusted Rand Index (ARI) measures agreement between cluster assignments and true labels while correcting for chance, with higher values indicating better correspondence^55^. Normalized Mutual Information (NMI) quantifies the amount of shared information between clustering results and true labels, with higher values indicating stronger agreement^59^. The V-measure represents the harmonic mean of homogeneity and completeness, evaluating how well clusters contain only members of a single class and whether all members of a class are assigned to the same cluster^57^. The Fowlkes-Mallows score measures pairwise similarity between clustering assignments and true labels, with higher values indicating better clustering performance^58^.

#### Using Tag data as a reference for call classification in long-term PAM data

To evaluate whether ground-truth tag data could serve as a reference for classifying FM calls in long-term PAM data, first the robustness of the UMAP projection was evaluated by partially excluding some of the tag data to serve as an evaluation set. For each species, one deployment was excluded from the Tag-dataset prior to building the embedding, representing 12% of all available fin whale calls and 15% of all available blue whale calls. UMAP projections were then generated using the remaining Tag data, and calls from the excluded deployments were projected into the resulting embedding space. Second, the FM calls in the Mooring-datasets were projected into a tag-derived UMAP space, using all available tag calls (Tag-RavenFeatures and Tag-DLFeatures). This means that the feature vectors from the excluded tag deployment and Mooring-datasets were transformed using the same 2-dimensional embedding (dimensionality-reduced) retrieved from the respective Tag-datasets, enabling direct comparison between the datasets.

For each FM call, the five nearest ground-truth tag neighbours in the embedding space were identified using Euclidean distance. The most frequent tag call type among these neighbours was assigned as the predicted label for the FM call. Calls whose nearest tag neighbour exceeded a Euclidean distance of 2 were classified as unassigned. To quantitatively assess the separability, Random Forest classification was performed using the Tag-RavenFeatures and corresponding Tag-DLFeatures. Like in the UMAP overlay analysis, one deployment per species was excluded from the Tag dataset and used as an independent test set, while the remaining Tag data served for model training. The classifiers were subsequently retrained using the complete Tag-dataset and applied to the unlabeled Mooring-dataset to predict species identity. Each Random Forest classifier was trained using 500 decision trees (n_estimators = 500), providing stable classification performance while minimizing sensitivity to random tree sampling.

## Supplementary Information

Below is the link to the electronic supplementary material.

Supplementary Material 1

## Data Availability

All data and code generated or analyzed in this study, including the tables used for analyses, are available from the GitLab repository: https://gitlab.awi.de/oza-sound-classifiers/raven-features-vs-ai-embedding-clustering. The long-term passive acoustic monitoring (PAM) recordings used in this study are publicly available at https://doi.org/10.1594/PANGAEA.966612, https://doi.org/10.1594/PANGAEA.966766, and https://doi.org/10.26179/5e6056035c01b.Blue whale tag data from Lewis et al., (2018) are available from the corresponding author upon reasonable request (https://doi.org/10.1098/rsos.180241). Fin whale tag data from Chile are currently unpublished.
