# Supplementary material for: Searching for separation between frequency-modulated calls of blue and fin whales
Source: Sci Rep. 2026 Sep 15;16:28772. doi: 10.1038/s41598-026-71077-1 (PMC13578311; doi:10.1038/s41598-026-71077-1)
Supplement: Supplementary file 1 [file 41598_2026_71077_MOESM1_ESM.docx]

Supplementary Material

Searching for separation between frequency-modulated calls of blue and fin whales.

**Svenja Wöhle,^1,*^ Clea Parcerisas^2,3^, Susannah Buchan^,4,5,6^, John Calambokidis^7^, Kathleen M. Stafford^8^ and Elena Schall^1^**

^1^ Alfred-Wegener-Institute Helmholtz Center for Polar and Marine Research, 27570 Bremerhaven, Germany

^2^ Flanders Marine Institute (VLIZ), 8400 Oostende, Belgium

^3^ Ghent University, IBME, 9052 Ghent, Belgium

^4^ Center for Oceanographic Research COPAS COASTAL, Universidad de Concepción, Casilla, Concepción, Región del Biobío, Chile

^5^ Facultad de Ciencias Naturales y Oceanográficas, Departamento de Oceanografía, Universidad de Concepción, Casilla, Concepción, Región del Biobío, Chile

^6^ Centro de Estudios Avanzados en Zonas Áridas (CEAZA), Raúl Bitrán, La Serena, Chile

^7^ Cascadia Research Collective, Olympia, WA, USA

^8^ Marine Mammal Institute, Oregon State University, Newport OR USA

* svenja.woehle@awi.de

**Figure A1.** Dimension reduction using an unsupervised UMAP approach on FM calls from the Mooring-DLFeatures-BioLingual.

**Table A1.** HDBSCAN clustering results and intrinsic clustering performance metrics for the Mooring-DLFeature-BioLingual dataset. The table summarizes the number and proportion of data points assigned to each cluster based on the unknown call types in the mooring dataset, together with intrinsic clustering evaluation metrics. Cluster entries show the number of data points, followed by the corresponding percentage (in parentheses) relative to the total number of data points in the respective dataset. Data points classified as noise by HDBSCAN are listed as unassigned. Intrinsic metrics include the Silhouette Score, Davies–Bouldin Index, and Calinski–Harabasz Index, which quantify cluster separation, compactness, and between-cluster dispersion in feature space, respectively.

| **Dataset** | **Cluster** | **No. total** | **Silhouette Score** | **Davies-Bouldin Index** | **Calinski-Harabasz (CH) Index** |
| --- | --- | --- | --- | --- | --- |
| *Mooring-*  *DLFeatures-*  *BioLingual* | unassigned | 7155 | 0.399 | 821.115 | 0.752 |
|  | 1 | 874 |  |  |  |
|  | 2 | 324 |  |  |  |

**Figure A2.** Dimension reduction using UMAP on the deep learning feature extraction model Biolingual on the Tag dataset. Panels on the left show UMAP projections generated using a supervised approach, while the panels on the right show unsupervised approaches. UMAPs A and B show only Tag data, C and D show the left-out deployment and projection approach, whereas E and F show the approach to project the Mooring-embeddings onto the Tag-embedding UMAP space. Unknown calls (*fm - lightgrey*), 40Hz-calls of fin whales (*40 - orange*), and D-calls of blue whales (*d - blue*) from the tag data are shown accordingly.

**Table A2.** Number and proportion of data points per cluster based on the two call types - 40Hz-calls of fin whales (40), and D-calls of blue whales (d) of the Tag-DLFeatures-BioLingual as determined by the HDBSCAN algorithm. Each entry shows the count of data points belonging to a given call type within a cluster, followed by the corresponding percentage (in parentheses) relative to the total number of data points in the respective cluster. Data points considered as noise by the algorithm are listed as unassigned in the cluster column.

| **Projection Type** | **Dataset** | **Cluster** | **No. of ‘40’ (%)** | **No. of ‘d’ (%)** | **No. total** |
| --- | --- | --- | --- | --- | --- |
| *unsupervised* | *Tag-*  *DLFeatures-*  *BioLingual* | unassigned | 21 (47.7) | 23 (52.3) | 44 |
|  |  | 1 | 45 (100) | 0 (0) | 45 |
|  |  | 2 | 0 (0) | 130 (100) | 130 |
|  |  | 3 | 0 (0) | 12 (100) | 12 |
| *supervised* | *Tag-*  *DLFeatures-*  *BioLingual* | unassigned | 0 (0) | 0 (0) | 0 |
|  |  | 1 | 45 (100) | 0 (0) | 45 |
|  |  | 2 | 21 (11.3) | 165 (88.7) | 186 |

**Table A3.** Extrinsic evaluation of clustering structure in the ground-truth Tag dataset for the BioLingual DLFeatures. Performance is assessed using the Adjusted Rand Index (ARI), Normalized Mutual Information (NMI), V-measure, and Fowlkes–Mallows score. These metrics quantify the degree of agreement between HDBSCAN clustering assignments and known call types (D- and 40Hz-calls), capturing different aspects of label-cluster correspondence including pairwise agreement, information overlap, and harmonic mean relationships between precision and recall.

| **Projection Type** | **Dataset** | **Adjusted Rand Index (ARI)** | **Normalized Mutual Information (NMI)** | **V-measure** | **Fowlkes-Mallows** |
| --- | --- | --- | --- | --- | --- |
| *unsupervised* | *Tag-*  *DLFeatures-*  *BioLingual* | 0.655 | 0.576 | 0.576 | 0.872 |
| *supervised* | *Tag-*  *DLFeatures-*  *BioLingual* | 1.0 | 1.0 | 1.0 | 1.0 |

**Figure A3.** Dimension reduction using supervised UMAP on the Tag-DLFeatures-BioLingual. The Mooring-DLFeatures-BioLingual are projected onto the corresponding Tag-UMAP spaces. Mooring calls are coloured according to the species attribution obtained using the k-nearest-neighbour (kNN) approach (left panel) and the Random Forest classifier (right panel). Fin whale 40Hz-calls are shown in orange, blue whale D-calls in blue, and remaining unknown calls (FM) in grey.

**Table A4.** Species attribution of FM calls in the Mooring-dataset obtained using the supervised UMAP-kNN and RandomForest approaches based on RavenFeatures and BioLingual-DLFeatures. Values indicate the number of calls with percentage in parentheses attributed as fin whale 40Hz-calls, blue whale D-calls, or unassigned.

| **Classification Approach** | **Dataset** | ***40Hz*** | ***D-call*** | ***Not attributed*** |
| --- | --- | --- | --- | --- |
| *UMAP + kNN* | *DLFeatures-*  *BioLingual* | 2661 (31.9%) | 4990 (59.7%) | 702 (8.4%) |
| *RandomForest* | *DLFeatures-BioLingual* | 4929 (59%) | 3424 (41%) | 0 (0%) |

**Figure A4.** Dimension reduction using supervised UMAP on the Tag-RavenFeatures and Tag-DLFeatures GoogleWhale and BioLingual. The Mooring-RavenFeatures and Mooring-DLFeatures are projected onto the corresponding Tag-UMAP spaces. Calls are coloured according to their measured Duration 90% values.
